# Selection of internal reference genes for normalization of reverse transcription quantitative polymerase chain reaction (RT-qPCR) analysis in the rumen epithelium

**DOI:** 10.1371/journal.pone.0172674

**Published:** 2017-02-24

**Authors:** Jose V. Die, Ransom L. Baldwin, Lisa J. Rowland, Robert Li, Sunghee Oh, Congjun Li, Erin E. Connor, Maria-Jose Ranilla

**Affiliations:** 1 Genetic Improvement of Fruits and Vegetables Laboratory, US Department of Agriculture, Agricultural Research Service, Beltsville, Maryland, United States of America; 2 Animal Genomics and Improvement Laboratory, US Department of Agriculture, Agricultural Research Service, Beltsville, Maryland, United States of America; 3 Dept. of Computer Science & Statistics, Jeju National University, Jeju City, Jeju Do, S. Korea; 4 Departamento de Producción Animal, Universidad de León, León, Spain; Universitat de Lleida, SPAIN

## Abstract

The rumen is lined on the luminal side by a stratified squamous epithelium that is responsible for not only absorption, but also transport, extensive short-chain fatty acid (SCFA) metabolism and protection. Butyrate has been demonstrated to initiate the differentiation of the tissue following introduction of solid feed to the weaning neonate as well as affecting the metabolism of other nutrients and absorption of nutrients in *in vitro* experiments. The objective of the present study was to validate expression stability of eight putative reference genes bovine rumen, considering the intrinsic heterogeneity of bovine rumen with regard to different luminal characteristics due to direct infusion of butyrate to double the intra-ruminal content of the rumen liquor. Our focus was on identifying stable reference genes which are suitable to normalize real-time RT-qPCR experiments from rumen samples collected from clinical assays, irrespective of localization within the organ and the across physiological state. The most stably expressed genes included: *ACTB*, *UXT*, *DBNDD2*, *RPS9*, *DDX54* and *HMBS*. Their high stability values suggest these reference genes will facilitate better evaluation of variation of across an array of conditions including: localization within the rumen, differences among cattle fed an array of rations, as well as response to development in the weaning animal. Moreover, we anticipate these reference genes may be useful for expression studies in other ruminants.

## Introduction

Given the unique placement of the organ in the gastrointestinal tract of the ruminant animal, the ruminal epithelium plays a vital and unique role in its health and productivity. Moreover, the ruminal epithelium is not simply a barrier to nutrient diffusion, but through the metabolism of short chain fatty acids (SCFA), primarily butyrate, acts to maintain the integrity of metabolite concentration gradients. In addition, ruminal SCFA metabolism protects against potentially detrimental decreases in blood pH [1,2]. Acetate, propionate and butyrate are metabolized to different extents by the ruminal epithelium. The impact of butyrate on the tissue is actively being investigated as a putative on-farm additive to enhance rumen development in weanling animals. Factors contributing to variation in feed efficiency among dairy cattle are not well understood, but likely include differences in basal energy requirements, levels of tissue metabolism, stress, and physical activity, and differences in nutrient digestibility or metabolism (e.g., during rumen microbial fermentation: [3]). Furthermore, these physiological factors likely have a genetic component [4]. Investigations into the molecular and genetic/epigenetic aspects of feed efficiency are needed to develop management strategies and selective tools for enhancing nutrient use efficiency by dairy cattle.

Visceral tissues represent 8–12% of body mass, yet account for a highly variable, and disproportionate amount of energy and nutrient use in ruminants (50–60% of total body nutrient use [5]). To date, our understanding of regulation of development of these organs is limited, in part, due to the inability to assess temporal changes in the gastrointestinal (GI) tract in vivo. Use of biopsy approaches can at a minimum provide invaluable information regarding the expression of genes under the influence of an array of luminal conditions.

Existing research on this subject was conducted *in vitro* using cell lines and short-term culture [6,7,8], or *in vivo* using experimental treatments and slaughter [8,9], combined with cell culture. Using biopsy procedures developed previously permits examination of the timeline of intestinal adaptive responses *in vivo* and enables critical sample collection without slaughter.

We sought to evaluate different reference genes for their potential use as internal normalization controls in gene expression measurements of rumen epithelial tissue from ontogenic studies *in vivo* to assess potential for luminal factors to impact tissue gene expression. In order to reduce the likelihood that our candidate genes exhibited regulated covariation, we chose a group of eight references with varied roles in cellular metabolism and molecular functions such as cytoskeletal structure (β-actin, *ACTB*), protein interactions (dystrobrevin binding protein, *DBNDD2*), ribosome biogenesis (DEAD box polypeptide 54, *DDX54*), glucose metabolism (glyceraldehyde-3-phosphate dehydrogenase, *GAPDH*), general metabolism (hydroxymethylbilane synthase, *HMBS*), matrix organization and collagen formation (cyclophilin B, *PPIB*), ribosomal structure (ribosomal protein S9, *RPS9*), and signaling pathways (prefoldin-like chaperone, *UXT*).

We attempted to (1) address each critical issue that needs to be carefully considered in a qPCR assay, and (2) identify a highly stable set of reference genes for butyrate-rumen-based experiments. Our results demonstrate highly stable values thus they may serve as useful guidelines or as a foundation point for reference gene selection for expression studies in other ruminants.

## Material and methods

All procedures involving animals were approved by the ARS Northeast Area Beltsville Location Institutional Animal Care and Use Committee protocol number 12–008.

### Animals and treatments

Holstein dry cows (n = 6) were surgically fitted with rumen fistulae using established methods. After recovery, cows were housed in tie stalls with *ad libitum* access to water and were maintained on the standard dry cow ration for the entirety of the experiment. Cows were allowed exercise daily, which was limited to 1 h/d during the infusion portion of the experiment. Ruminal infusion of butyrate was initiated immediately following 0h sampling (baseline controls) and thereafter continued for 168h at a rate of 5.0L/d of a solution representing > 10% of daily anticipated metabolizable energy intake in a buffered saliva solution (pH 7.0; 3.8% KHCO_3_, 7.3%NaHCO_3_) as a continuous infusion. Following the 168h infusion, infusion was terminated and cows were maintained on the dry cow ration for an additional 168h for sampling. Rumen epithelial samples were serially collected via biopsy through rumen fistulae at 0, 24, 72 and 168h of infusion, and 168h post infusion. Rumen epithelial samples were saved in RNAlater (Life Technologies, Grand Island, NY) and stored in −80C until RNA extraction.

### Sampling

Rumen biopate (papillae) were collected by grab biopsy from the ventral cranial sac of the rumen and immediately processed for RNA collection. Briefly, rumen papillae samples were washed in large volumes of physiological saline to remove visible debris before being stabilized in RNAlater according to the manufacturer’s instructions, and stored at −80°C until RNA extraction. Tissues were homogenized in Qiazol Lysis Reagent (Qiagen, Valencia, CA) and total RNA was extracted using the RNeasy Mini Kit (Qiagen) with on-column DNase digestion.

RNA concentration was determined by measuring the optical density at 260 nm using a Nanodrop ND-1000 spectrophotometer (NanoDrop Technologies, Wilmington, DE). RNA quality was assessed by combining information from several control steps. First, purity was inferred from the absorption ratios using the NanoDrop. Only the samples with A260/A280 absorption ratios from 1.8–2.2 and A260/A230 > 1.5 were used in the analysis. Then RNA integrity was assessed by microcapillary electrophoresis with an Experian RNA StdSens Chip and Experion Bioanalyzer (Bio-Rad Laboratories, Hercules, CA), showing rRNA subunits with 18S/28S peaks on the virtual gel and electropherograms. Only RNA samples with electropherograms of high quality and RQI ≥ 8 were used for further study. All samples were pure and free from protein and organic pollutants derived from the RNA extraction. After performing RNA quality controls, RNA was deemed as suitable for qPCR analysis.

### First strand cDNA synthesis and genomic DNA contamination

RNA samples were adjusted to the same concentration, measured on NanoDrop and adjusted again in order to equalize the RNA input in the subsequent reverse-transcription reaction. Then, RNA (1 μg) was reversed-transcribed with a blend of oligo(dT) and random hexamers primers using the iScript cDNA synthesis kit (Bio-Rad), according to the manufacturer’s instructions. The cDNAs were diluted to a final volume of 200 μl. Absence of genomic DNA contamination in RNA samples was tested by PCR using primers designed to amplify a 716-bp fragment (spanning exons 4, 5 and 6) of the *β-actin* gene (AC_000182exon4: 5’-GGCTACAGCTTCACCACCAC-3’, AC_000182exon6: 5’-ACTCCTGCTTGCTGATCCAC-3’). A quantity equivalent to the cDNA used as template in the subsequent amplification PCR (i.e., 5 ng of gDNA) was used as positive control. Clear distinguishable 496-bp bands were found over the cDNA samples. No cDNA showed the 716-bp fragment. As second way to test the gDNA contamination, we designed (if possible) and checked reference genes primers to generate amplicons with different length from gDNA or cDNA (**Table 1**). **S1 Fig.** shows those primer pairs positions mapped to their respective gene sequence using Bioconductor and the IRanges package [10,11]. After performing gDNA control, the cDNA samples were deemed as suitable for qPCR analysis.

**Table 1:** Description of reference genes and primer sequences. Primer PCR efficiency and PCR Tm product data represent mean values ± SE (n = 30). PCR efficiencies (E) calculated according to the equation (1 + E) = 10^slope^. Free energy for potential secondary structures in the amplicon is shown (ΔG, kcal/mole).

Gen Id	Symbol	Name	Primer Sequence (5'-3')	Exon	ΔG (kcal/mole)	PCR E	PCR product cDNA/gDNA	PCR product Tm (°C)
NM_174152	*PPIB*	cyclophilin B	^4806^CGGCAAAGTTCTAGAGGGCA	4	0.55	1.06±0.08	85/830	83.18±0.25
			^5636^CACGTCCTTCAGAGGCTTGT	5				
NM_173979	*ACTB*	β-actin	^2714^AGTACTCCGTGTGGATTGGC	5	0.59	1.00±0.04	78	81.22±0.28
			^2791^ACTCCTGCTTGCTGATCCAC	5				
NM_001046207	*HMBS*	hydroxymethylbilane synthase	^3468^ACCGCGCTCTCTAAGATTGG	4–5	0.34	1.01±0.06	66/155	80.50±0.15
			^3622^CCTCTCCAAAGCATGCTCCA	5				
NM_001034034	*GAPDH*	glyceraldehyde-3-phosphate dehydrogenase	^3374^GTCATCCCTGAGCTCAACGG	7	0.07	1.00±0.05	69/155	79.57±0.22
			^3528^AACAGACACGTTGGGAGTGG	8				
NM_001037471	*UXT*	prefoldin-like chaperone	^6090^CACATGTTGCTAGAGGGGCT	5–6	0.5	1.01±0.06	66/281	81.18±0.28
			^6370^TCAGTGCTGAGTCTCTGGGA	6				
NM_001130748	*DBNDD2*	dystrobrevin binding protein	^2270^GTGGAGCTTATCGACCTGGG	2	0.82	0.99±0.07	74	82.00±0.19
			^2343^GGAGTTGGTGGAGGGTCTTC	2				
XM_002694516	*DDX54*	DEAD box polypeptide 54	^15818^AAGAAGCGGTTTGTGGGACA	18	0.9	0.99±0.06	74	84.43±0.17
			^15891^CTGATGTAGCGGCCACTCTC	18				
BC148016	*RPS9*	ribosomal protein S9	^6113^TGCTGGATGAGGGCAAGATG	3	0.98	1.02±0.03	75	84.37±0.22
			^6187^GCAGGCGTCTCTCCAAGAAA	3				

### Selection of internal control genes

We selected several candidate reference genes based on the following criteria: (i) sequences should encode for proteins that perform varied roles in cellular metabolism with different molecular functions in order to minimize the effect of co-regulation, (ii) sequences which had previous been assessed for stability in, at least, slightly similar biological contexts, and (iii) because we aimed to perform several *in silico* controls on the primer pairs and amplicons, sequences with reliable and accessible prior information. However, we designed the primers *de novo* to meet our quality control criteria. Thus, qPCR primers were designed with the following criteria: lengths of 20 nucleotides, Tm of 60±1°C and GC content of 50–60% yielding PCR amplicon lengths of 60–90 nucleotides. We chose candidate sequences from studies aimed to measure mRNA abundance of rumen epithelial genes [12], genes involved in ruminal development [13], glucose transporters in the mammary gland [14] or orthologs of stable references previously used in different tissues in the phylogenetically bovine closely related *Capra hircus [15,16]*. All qPCR primers were tested for specificity using NCBI’s BLAST software [17]. For the prediction of secondary structure of the amplicons, we used the mfold Web Server with default settings of minimal free energy, 50 mM Na^+^, 3mM Mg^2+^, and annealing temperature of 60°C [18]. We chose primers that would yield amplicons with minimal secondary structures and melting temperatures that would not hamper annealing. We ultimately selected 8 primer pairs that met our quality controls. Designed primers were synthesized by Integrated DNA Technologies (Coralville, IA). **Table 1** shows the overall mean real-time qPCR amplification efficiency of each primer pair (E) estimated from the data obtained from the exponential phase of each individual amplification plot and the equation (1+E) = 10^slope^ using LinReg software and the criteria of including three to five fluorescent data points with R^2^≥0.995 to define a linear regression line [19]

### Real-time qPCR assays

PCR reactions were carried out in an IQ5 (Bio-Rad) thermal cycler using SsoAdvanced™ Universal SYBR® Green Supermix (Bio-Rad) to monitor dsDNA synthesis. Reactions contained 1 μl of the diluted cDNA as a template and 0.150 μM of each primer in a total volume reaction of 20 μl. Master mix was prepared and dispensed into individual wells using electronic Eppendorf Xplorer® multipipettes (Eppendorf AG, Hamburg, Germany). The following standard thermal profile was used for all qPCRs: polymerase activation (95°C for 3 min), amplification and quantification cycles repeated 40 times (95°C for 30 sec, 60°C for 1 min). The specificity of the primer pairs was checked by melting-curve analysis performed by the PCR machine after 40 amplification cycles (60 to 95°C) and is shown in **S2 Fig.** Fluorescence was analyzed using iQ5 2.1 standard optical system analysis software v2.1 (Bio-Rad). All amplification plots were analysed using a base line threshold of 30 relative fluorescence units (RFU) to obtain C_q_ (quantification cycle) values for each gene-cDNA combination.

### Data analysis

To determine which reference genes were best suited for transcript normalization in bovine rumen, we first used the statistical algorithm geNorm [20]. In a second approach, the coefficient of variation of normalized relative expression levels was calculated based on the formulas (formula 11, 13, 15, 17, 18, 19 and 20) described in the qBase software [21]. In short, C_q_ values were incorporated into an in-house developed R script [22] and transformed into relative quantities (RQs) using the efficiency of each primer pair and the sample with the lowest C_q_ as a calibrator. Then, a sample-specific normalization factor (NF) was estimated as a geometric mean of RQs for the candidate genes. Finally, the mean coefficient of variation (CV) for all reference genes was calculated as the arithmetic mean of the CV estimated from the different reference genes.

### Assessment of normalization

To validate the reliability of the results we addressed the influence of the choice of one reference gene on the interpretation of output data. First, we estimated two normalization factors (determined by calculating the geometric mean of the two best-scored and the two worst-scored reference genes) and we plotted the normalized relative quantities for two genes over the time-course experiment and for all samples. As a second approach to validate the stable expression of the references, the expression level of a gene of interest encoding a regulator of chromosome condensation (*RCC2*, NM_001101911F: 5’-CCTGTGGGGCTGAATTCAGT-3’; NM_001101911FRv: 5’-GTTGTGTCCCAGCTGACCAT-3’) was quantified at two different time points (0–14 days) and was normalized to those normalization factors. Finally, we addressed the normalization based on the use of one single reference gene. Expression level ratios for *RCC2* were quantified at days 0 and 14 normalized to each of the reference genes using the delta-delta method modified by the efficiency correction as described by [23].

## Results and discussion

Previously, in lactating cattle, similar infusion approaches elicited changes in a wide array of gene families [24]. Prior experiments, in lactating cattle [24,25], demonstrated that butyrate concentration in the rumen attained pleateau by 168 h, and had largely returned to pre-infusion concentrations by 168 post removal. Thus, changes reflecting new expression levels are best assessed at these time points while changes occurring in 24 and 48 h are more likely to reflect gene expression changes in response to the luminal environment and thus, will likely lend insight into mechanism of action accounting for the response.

### Quality controls and overall gene expression

Due to the difficulty in comparing various data sets related to perturbation of rumen SCFA concentrations (e.g., sub acute ruminal acidosis models, direct infusion, physiological status[8]) and putative power of RNAseq as a tool for assessment of transcriptomic changes in response, a validated set of reference sequences will enhance future interpretations. We anticipated a need for a solid validation of a select set of potential reference genes using two bioinformatics algorithms: geNorm and qBase. Factors known to affect the reliability of gene expression data such as RNA quality, DNase treatment, two-step RT-qPCR, the use of the same RT master mix that generated one cDNA batch, primer design keeping in mind the presence of secondary structures in the primer as well as in the amplicon, PCR efficiency correction, and non-specific amplification were closely controlled during the experiment. High-quality total RNA was obtained and evaluated by microcapillary electrophoresis and absorbance ratios. To exclude genomic DNA contamination, a fragment of *ACTB* cDNA (GenBank accession number NM_173979) was amplified with primers designed to span two introns to generate bands clearly distinguishable between cDNA and gDNA. No cDNA showed the 716-bp fragment characteristic from gDNA. Also, for some sequences was possible to design assays with primers spanning an intron. Primer position on the sequence, number of exon and different sizes for amplicons that we used to confirm that our material was free of gDNA contamination is shown in **Table 1**. After performing the *in silico* quality controls on primer pairs and amplicon sequences described in the Materials and Methods, 8 primer pairs from these genes were retained for the experimental analysis. Amplification specificity of all SYBR Green assays was confirmed by running a dissociation protocol using incremental temperatures to 95°C. The melting curve analysis showed that each of the 8 primer pairs amplified a single product.

**Fig 1:**
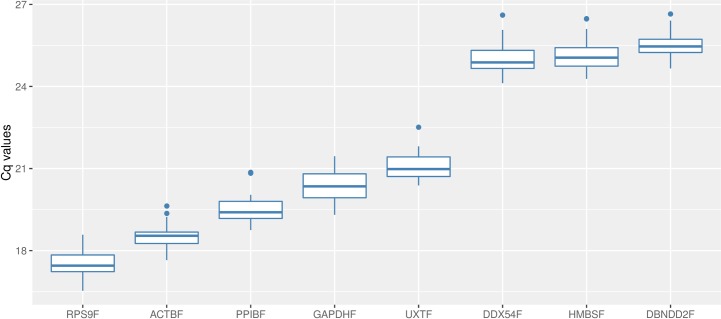
Boxplot of Cq values. Variations of the quantification cycle are shown for all examined reference genes sorted by expression range (n = 30). Each box represents the 50% quartile; the median is figured as the line in each box; whiskers depict the 25% minimum and maximum range of the Cq value.

Real-time qPCR reactions provided data within variable Cq value ranges for the 8 references. *RPS9F* had the lowest mean Cq value (17.51), while *DBNDD2F* had the highest one (25.52), showing an expression level approximately >250-fold lower than *RPS9F* (**Fig 1**). Preliminary analysis of data showed that individual reference genes had similar Cq values variation across all studied samples (~2 cycles). The smallest variation was observed for *ACTBF* (1.98 cycles), while *DDX54F* was the gene with the widest range (2.49 cycles). All assays were found to have high PCR efficiency of amplification. Amplification efficiencies varied from 0.988 for *DDX54F* to 1.057 for *PPIBF* indicating comparable amplification in the 30 cDNAs tested (**Table 1**).

### Stability analysis

**Fig 2:**
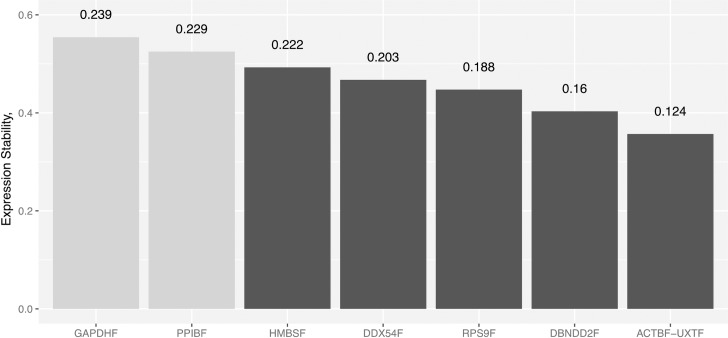
geNorm ranking of 8 reference genes from bovine rumen samples. Vertical numbers at the top indicate the CV values of the reference genes involved in the normalization. References showing highly stable expression (*M* values < 0.5) are represented as black bars.

To analyze the stability of the expression and identify suitable reference genes, we used the statistical algorithm geNorm [20]. The geNorm software tool represents the most commonly used algorithm to evaluate candidate reference genes and has established itself as the *de facto* standard method [26,27]. The program defines a stability measure (*M*) as the average pairwise variation between a gene and all other reference genes. The stability measurement relies on the principle that the expression ratios of two ideal internal genes are identical in all samples, regardless of the experimental condition. The lower the *M* value, the more stably expressed is the gene. In a second approach, the CV for all reference genes were determined based on the equations defined by the qBase framework [21]. **Fig 2** shows the ranking of the genes tested in our samples according to their *M* and CV values. In our material, without exception, stability values were much lower than the default threshold of 1.5 defined in the algorithm. In fact, none of the tested candidate reference genes exceed the *M* value = 0.6. In addition, 6 out the 8 references show *M* values < 0.5 indicating that their stable expression makes them highly suitable as internal references in rumen gene expression analysis. geNorm algorithm indicated that *ACTBF* and *UXTF* are the most stable genes (*M* = 0.36), whereas the lowest stability value was recorded for the *GAPDH* gene (*M* = 0.55). Genes with the values inside the optimal range for homogeneous sample panels (*M* and CV values lower than 0.5 and 25% respectively) according to [21] were the most stably expressed and included: *ACTBF*, *UXTF*, *DBNDD2F*, *RPS9F*, *DDX54F* and *HMBSF* (*M* = 0.49 and CV = 0.222).

### Validation of reference genes

**Fig 3:**
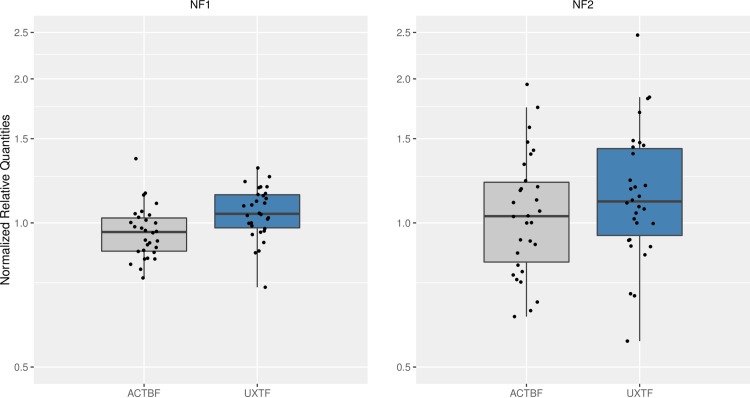
Evaluation of reference genes for bovine rumen. The relative quantities of genes *ACTBF* and *UXTF* were normalized for each sample against the 2 best-scored (NF_1_) and the 2 worst-scored references (NF_2_). Y-axis is shown in logarithmic scale.

To test the putative reference genes identified above, we estimated the normalized relative quantities of the two best reference set across the samples compared to their overall geometric mean. The values were closely distributed around 1-fold and only a small difference between samples could be detected (1.77-fold on average) when we used the two best-scored references as calibrators (**Fig 3**). When we normalized the expression of the same genes against the geometric mean of the references with the highest *M* value, the values were also closely distributed around 1-fold. However, we clearly observed average differences of over 3-fold between samples (3.70-fold). This illustrates that the selection of the references matters and how in the second case, by using a references pair that it is not the optimal, we add technical variation during the normalization step instead of removing variation (**Fig 3**).

**Fig 4:**
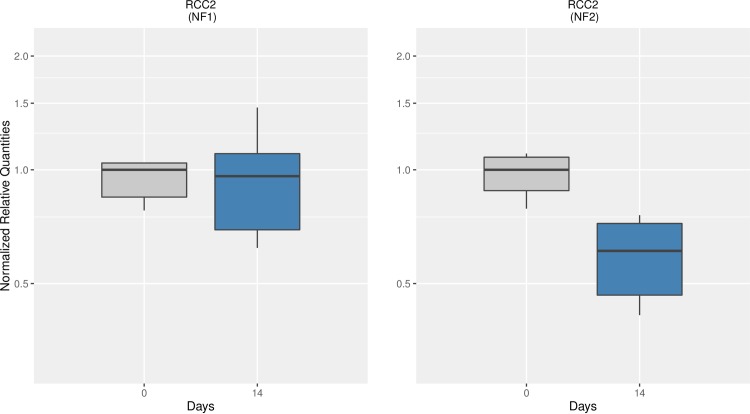
Normalized relative quantities of *RCC2* over days 0 and 14. Data were normalized for each sample against the 2 best-scored (NF_1_) and the 2 worst-scored references (NF_2_). NRQ were rescaled to day 0 that was arbitrarily set as 1.

In a second attempt to validate the stability of the references, we monitored the mean expression of one target gene at days 0 and 14. Normalized relative quantities of the regulator of chromosome condensation *RCC2* were obtained using two normalization factors: again, the use of the 2 best-scored references (NF_1_) and the use of the 2 worst-scored references (NF_2_). **Fig 4** shows the relationship of the transcript levels between those days. Fourteen days after butyrate infusion, we did not observe any difference in the amount of *RCC2* transcripts in the rumen epithelium when the normalization factor NF1 was used. However, when we measured the transcriptional activity of the same gene, on the same subjects, at the same time-points but using the NF2, the mRNA expression levels of *RCC2* decreased about ~ 2-fold. The shift in the average expression levels is due to solely the selection of the reference genes to build the normalization factor. Therefore, selection of references without previous knowledge of their expression stability values are likely to create technical artifacts due to their intrinsic variation.

### Single reference-based normalization

**Fig 5:**
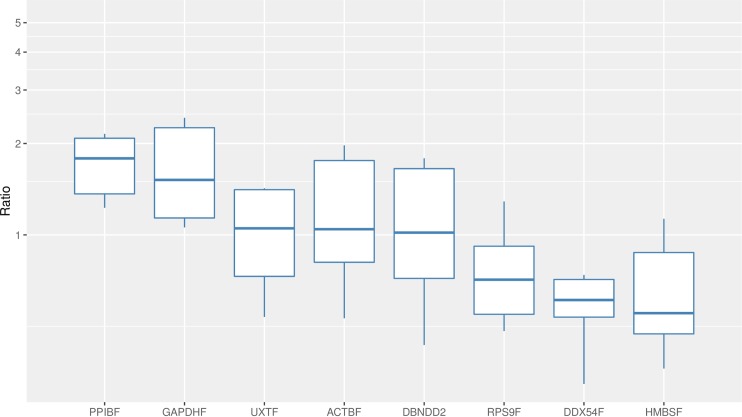
Normalization of *RCC2* mRNA expression at 0 and 14 days after butyrate infusion using 1 single reference gene. The X-axis shows the internal gene that was used for estimating each ratio. Y-axis is shown in logarithmic scale.

One of the most intriguing issues in the field of qPCR relates to normalization using only one single non-validated reference gene. Despite the vast number of publications showing that the use of a single reference yields unreliable data, this approach is still routinely used in animal, plant and biomedical research. After the publication of the MIQE guidelines, Short [28] noted that only 10.5% of qPCR analyses published in three leading high-impact journals used more than one single reference gene. The high stability expression levels found in our study (low *M* and CV values) might lead one to think that our results are not compromise by the use of one single reference gene during the normalization. In order to test this hypothesis, we quantified again the ratio of *RCC2* at days 0 and 14, but this time we normalized that level relative to each of the 8 stable reference genes identified in the study. Normalization of *RCC2* mRNA with the various references showed that the ratios obtained were highly different depending on the reference gene used. It is noteworthy to mention that we found even contradictory results, as some ratios suggest up-regulation of *RCC2* (ratio > 1-fold) whereas some ratios indicate down-regulation over the same period of time (ratio < 1-fold). The average *RCC2* mRNA ratio (0–14 days) was 1.31-fold but it ranged from being down-regulated (1.75-fold) to being up-regulated (3.17-fold), depending only on the normalizer chosen (**Fig 5**). Thus, even working with highly stable genes across a given dataset, as in our case here, we found that differences in expression levels of ~5.6-fold can be generated based on only the choice of the single reference gene. It is likely that those differences will be greater if the reference has not been validated or worse, if it is a non-stable reference gene. With this analysis we demonstrate that the use of one non-validated reference gene is also a source of introduction of technical variation, may create confounding variation and may lead to misinterpretation of our conclusions. This also points out the highly improbable chance that small differences in gene expression, such as those *a priori* expected for a transcription factor or other regulators, could be detected based on the sole use of one reference gene.

## Conclusions

Increasingly there is interest in understanding the impact of visceral organ impact, including gastrointestinal tissues, on animal biology across agricultural species. Therefore identifying a stable set of quality reference genes which are suitable to normalize real-time RT-qPCR experiments from rumen epithelial tissue samples is needed. We have found that the most stably expressed genes were *ACTB*, *UXT*, *DBNDD2*, *RPS9*, *DDX54* and *HMBS* and their high stability indicate that these reference genes will facilitate better evaluation of variation across an array of physiological conditions including: localization within the rumen, differences among cattle fed an array of rations, as well as response to development in the weaning animal. Moreover, we anticipate these reference genes may be useful for expression studies in other ruminants.

## Supporting information

S1 Fig Position of reference genes exons and primer pairs. X-axis represent fragment length in bp. Bottom row on X-axis shows number and position of exons. On top of exons, primer sequences forward and reverse are shown. Red color denotates forward primer spanning an intron.
(TIF)

S2 Fig Melting curve analysis to determine the specificity of the amplicons. (TIF)
