# Supplementary figures and images for: Selection of internal reference genes for normalization of reverse transcription quantitative polymerase chain reaction (RT-qPCR) analysis in the rumen epithelium
Source: PLoS One. 2017 Feb 24;12(2):e0172674. doi: 10.1371/journal.pone.0172674 (PMC5325532; doi:10.1371/journal.pone.0172674)

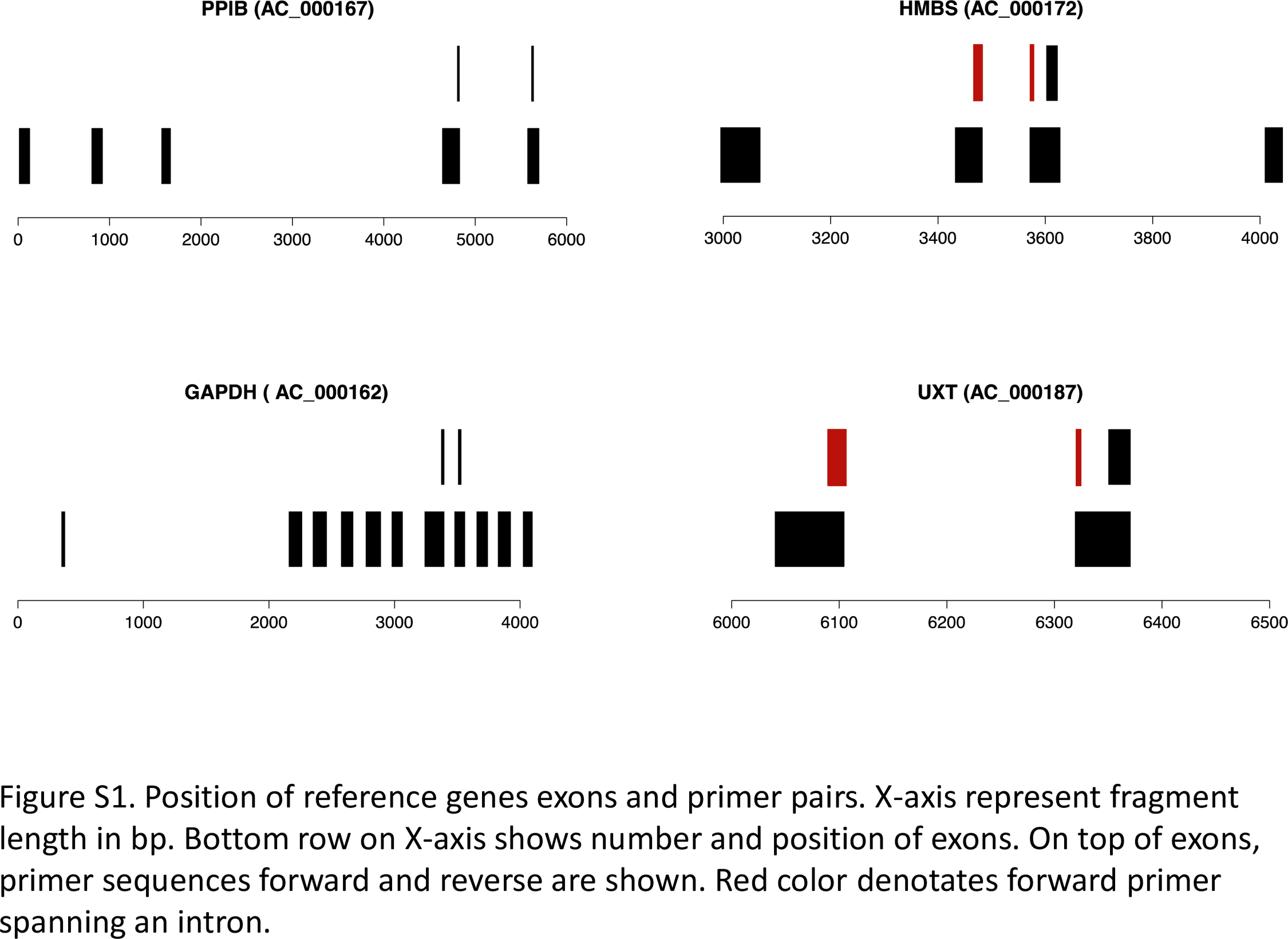

Supplement: S1 Fig — X-axis represent fragment length in bp. Bottom row on X-axis shows number and position of exons. On top of exons, primer sequences forward and reverse are shown. Red color denotates forward primer spanning an intron. (TIF) [file pone.0172674.s001.tif]

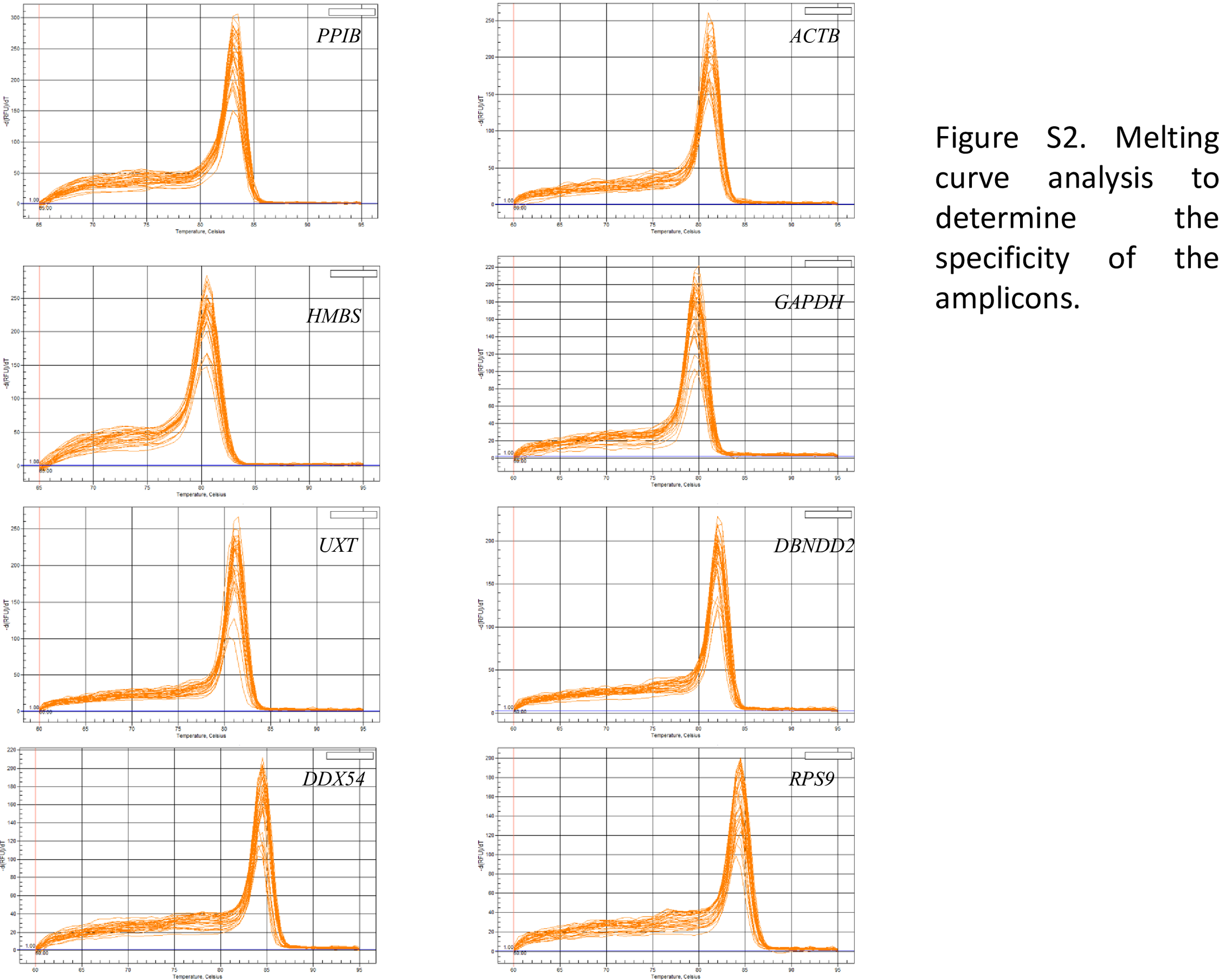

Supplement: S2 Fig — (TIF) [file pone.0172674.s002.tif]
